# Roles of Noncoding RNA in Reproduction

**DOI:** 10.3389/fgene.2021.777510

**Published:** 2021-12-09

**Authors:** Chaofan He, Kaixian Wang, Yuanyuan Gao, Chen Wang, Leina Li, Yaping Liao, Ke Hu, Meng Liang

**Affiliations:** School of Life Science, Bengbu Medical College, Bengbu, China

**Keywords:** miRNA, lncRNA, piRNA, spermatogenesis, follicular development, reproductive disease

## Abstract

The World Health Organization predicts that infertility will be the third major health threat after cancer and cardiovascular disease, and will become a hot topic in medical research. Studies have shown that epigenetic changes are an important component of gametogenesis and related reproductive diseases. Epigenetic regulation of noncoding RNA (ncRNA) is appropriate and is a research hotspot in the biomedical field; these include long noncoding RNA (lncRNA), microRNA (miRNA), and PIWI-interacting RNA (piRNA). As vital members of the intracellular gene regulatory network, they affect various life activities of cells. LncRNA functions as a molecular bait, molecular signal and molecular scaffold in the body through molecular guidance. miRNAs are critical regulators of gene expression; they mainly control the stability or translation of their target mRNA after transcription. piRNA functions mainly through silencing genomic transposable elements and the post-transcriptional regulation of mRNAs in animal germ cells. Current studies have shown that these ncRNAs also play significant roles in the reproductive system and are involved in the regulation of essential cellular events in spermatogenesis and follicular development. The abnormal expression of ncRNA is closely linked to testicular germ cell tumors, poly cystic ovary syndrome and other diseases. This paper briefly presents the research on the reproductive process and reproductive diseases involving ncRNAs.

## Introduction

With the development of genome-wide sequencing technology and high-throughput sequencing technology, it has been found that about 93% of the DNA sequences in the human genome can be transcribed into RNA, but only about 2% of the DNA sequences eventually encode proteins, with 90% of the DNA sequences being transcribed into ncRNAs (Consortium et al., 2007; Consortium, 2012). According to the length, a large number of noncoding RNA in these cells can be classified into lncRNA (>200 nt) and small noncoding RNAs (sncRNA, <200 nt). There are many types of sncRNAs, which can be divided into constituent and regulatory types. Regulatory sncRNAs include miRNAs and piRNAs (Chen et al., 2019a). In the past 20 years, great progress has been made in the study of the role of small noncoding RNAs in cellular life, but the study of lncRNAs has attracted people’s attention in recent years. As an important member of the gene regulatory network, previous gene transcriptional “noise” plays a very important role in the physiological and pathological processes of cells (Yap et al., 2010; Wang and Chang, 2011). Studies have demonstrated that noncoding RNA participates in the regulation of spermatogenesis and oogenesis (Garcia-Lopez et al., 2015). In recent years, more and more evidence has shown that ncRNAs are regulators of many biological processes and participate in multiple levels of gene expression regulation in the form of RNA in spermatogenesis and follicular development, such as genomic imprinting, cell proliferation, cell differentiation and meiosis (Bernard et al., 2015; Taylor et al., 2015; Robles et al., 2019).

Spermatogenesis is a process through which male animals produce male gametes continuously. That is, spermatogonia stem cells (SSCs) undergo a series of strictly regulated physiological progress to constitute spermatozoa. Spermatogenesis consists of the following stages: stem-cell mitosis produces spermatocytes which undergo two meiosis to produce haploid round sperm cells, which eventually become mature sperm (Griswold, 2016). Each stage of spermatogenesis is precisely monitored by a variety of factors. Therefore, elucidating the molecular mechanism of spermatogenesis can better elucidate the genetic regulation of male germ cell development. More significantly, it lays a solid foundation with which to diagnose and treat male infertility.

The ovary is the female reproductive organ. Its main functions consist of secreting sex hormones and producing mature eggs. The follicles, the fundamental unit of the ovary, are comprised of the oocytes located in the middle, and the surrounding granuloma cells and membrane cells. According to the different stages of development, follicles can be divided into primordial follicles, primary follicles, secondary follicles, astral follicles and preparatory follicles. In fact, only a few follicles can develop to the ovulation stage and ovulate, and most follicles move towards atresia at different stages of development. Maturation or atresia of an egg during follicular development is formally regulated by a variety of factors, including paracrine and autocrine factors in the endocrine and ovary, and cellular communication between oocytes and granulosa cells, and between granuloma cells and granulosa cells (Son et al., 2011; Emori and Sugiura, 2014). Therefore, the study of follicular development determines significance for the diagnosis and treatment of female infertility.

In recent years, abundant hallmark studies in various organisms show that non-coding RNA directly and indirectly regulate the reproductive system. For example, it interacts with proteins and regulates their functions (Statello et al., 2021), regulates histone modifications (Greco and Condorelli, 2015), promotes mRNA degradation (Beavers et al., 2015) and silence transposons (Reuter et al., 2011), etc. This review will focus on how ncRNA regulates spermatogenesis and follicular development, as well as the role of ncRNA in reproductive diseases.

## microRNAs

### microRNA Functions

MicroRNAs (miRNAs) are a class of noncoding RNAs with regulatory functions discovered in eukaryotes in recent years. They are primarily involved in the regulation of genes at the post-transcriptional level. The regulatory functions of miRNAs are indispensable, and they have recently been found to play an important regulatory role in immune diseases, cancer and reproductive diseases (Yao et al., 2010a; Fleshner and Crane, 2017; Lai et al., 2019). miRNAs are also engaged in gametogenesis during sexual reproduction.

**FIGURE 1:**
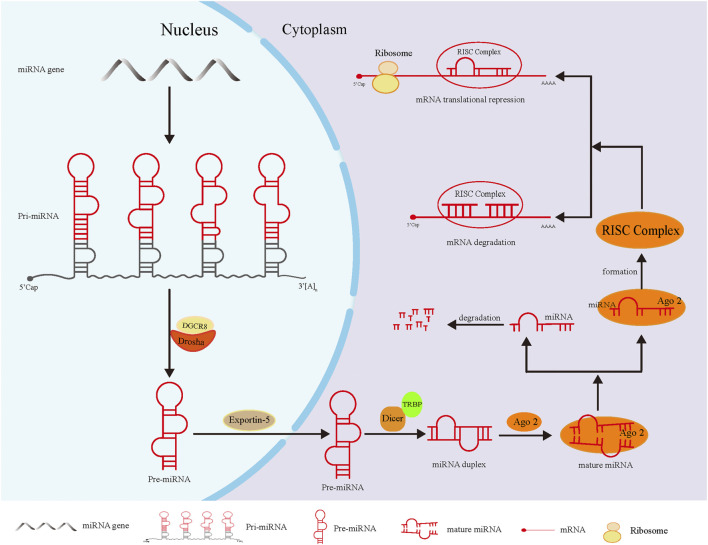
The biosynthesis of miRNA. In the nucleus, pri-miRNA, the primary transcription product of miRNA gene, was cleaved by Drosha-DGCR8 complex into hairpin precursor miRNA. After preliminary shearing, the pre-miRNA was transferred to the cytoplasm under the action of the transporter Esportin-5. In the cytoplasm, the pre-miRNA was further cleaved by Dicer enzymes that bound to TRBP (double-stranded RNA-binding protein) to form miRNA duplex of mature length. Finally, the double-stranded miRNA was loaded into Ago2 to generate mature miRNA. Subsequently, one chain of miRNA dissociated from the complex and degrades rapidly, and the remaining chain formed the RISC complex with Ago2, which led to degradation or translation inhibition of target mRNA.

The biosynthesis of miRNAs in animals (especially humans) has been preliminarily interpreted. First, the primary transcripts of miRNA genes (pri-miRNAs) are converted into precursor miRNAs (pre-miRNAs) in the nucleus by the RNase, Drosha. After the initial splicing, pre-miRNAs are transferred from the nucleus to the cytoplasm under the action of the transporter Exportin-5, and then further cleaved by another RNase, Dicer, to produce mature miRNAs. These mature miRNAs, together with other proteins, form the RNA-induced silencing complex (RISC), which causes the degradation of target mRNA or the inhibition of translation (Johanson et al., 2013; Wu et al., 2018). This process is shown in Figure 1.

### The Role of microRNAs in Spermatogenesis

miRNAs are special minor molecules of RNA, with a size of approximately 22 bps. With the development of deep sequencing technology, a large number of miRNAs have been discovered in human and mouse genomes, the miRbase database (https://www.mirbase.org/) revealed 2654 mature miRNAs in the human genome and 1978 miRNAs in the mouse genome (Griffiths-Jones et al., 2008). These miRNAs are highly conserved between species and have been reported to regulate over 30% of genes in the genome (Lewis et al., 2005). The establishment of a miRNA expression profile in male germ cells is an essential prerequisite for comprehensively exploring the biological function of miRNAs in spermatogenesis. Although the role of miRNAs in the development of male germ cells has not been fully studied, high-throughput expression profiles have found that a large number of miRNAs are selectively expressed in spermatogonia, pachytene spermatocytes, spermatozoa and mature spermatozoa (Yan et al., 2007; Moritoki et al., 2014). miRNAs are specifically expressed at all stages of spermatogenesis, suggesting involvement in almost every step of male germ cell differentiation.

Some studies conducted conditional knockout of Drosha and Dicer in spermatogenic cells of mice testis after birth, and found that the knockout mice were infertile due to impaired spermatogenesis, presenting oligospermia or azoospermia, suggesting that miRNA is involved in regulating spermatogenesis and plays a crucial role (Bernard et al., 2015).

Spermatogenesis originates from SSCs, and many miRNAs are involved in the mitotic proliferation of SSCs. In recent years, with the development of analytical techniques, hundreds of miRNAs have been discovered. A large number of miRNAs were found to be highly expressed in SSCs, such as clusters of miR-17-92 and miR-290-295. miRNA-20, miRNA-21 and miRNA-106a can regulate the self-renewal of SSCs (Niu et al., 2011; He et al., 2013b). The role of miRNAs in regulating the fate of SSCs has been confirmed, but the specific regulatory mechanism remains to be further studied. Some miRNAs are associated with regulating the proliferation and apoptosis of SSCs. For example, miR-204 regulates the proliferation of SSCs by targeting Sirt1 (Niu et al., 2016), while miR-34c can affect the apoptosis of SSCs (Li et al., 2013). miR-21 is involved in SSCs mitotic proliferation by regulating ETV5, a key gene that maintains SSCs self-renewal (Kotaja, 2014). miR-20 and miR-106a, which are preferentially expressed in SSCs, stimulate the proliferation of SSCs by targeting inhibition of STAT3 (signal transduction and transcriptional activator 3) and CCND1(cyclin D1). This is mainly because the silencing of STAT3 and CCND1 can stimulate the renewal of SSCs (He et al., 2013a). The above studies show that miRNAs have restricted expression during spermatogenesis, can regulate the proliferation of SSCs, participate in the regulation of gene transcription in the spermatogenesis and meiosis of germ cells, and play a regulatory role in maintaining the normal development of male germ cells.

There are also many miRNAs involved in meiosis, which regulate meiosis by acting on different targets and play an important role in spermatocyte differentiation. miR-10a, recently reported, also plays a critical role in mouse and human male germ cell development and spermatogenesis by regulating the meiosis process. miR-10a negatively targets RAD51, and its overexpression can lead to complete male sterility and meiosis stagnation (Gao et al., 2019). In addition, deletion of miR-871 and miR-880 also leads to meiosis stagnation in spermatogenesis, which inhibits Fzd4 gene expression by targeting the 3′-UTR of its target mRNA (Ota et al., 2019). Specific miR-34c and miR-34b-5p in pachytene spermatocytes and round spermatocytes also promote further development of spermatogenesis by inhibiting cell proliferation (Smorag et al., 2012; Wang et al., 2018b). miR-34b-5p regulates meiosis by targeting Cdk6. miR-34c affects meiosis by targeting Notch1, CDK4, MYC and other cell cycle regulators, and TGIF2 and Notch2 are direct targets of miR-34c (Bouhallier et al., 2010). In addition, miR-34c can also promote can also promote the apoptosis of male germ cells by targeting ATF1 (Liang et al., 2012).

miRNAs have become novel key factors in post-transcriptional regulation of male germ cells and are widely involved in various developmental stages of spermatogenesis (Taylor et al., 2015). Currently, although many miRNAs have been identified to play an important role in spermatogenesis and their functions and regulatory mechanisms have been annotated, the roles and potential mechanisms of many miRNAs remain unknown. In the future, it will become an important research direction to reveal the role of specific miRNAs in spermatogenesis. These results will contribute to understanding the etiology of male infertility and provide a theoretical basis for exploring the prevention and precise treatment of male infertility and other diseases.

### The Role of microRNAs in Follicular Development

As embryos of mice with Dicer1 allele deletion died, Otsuka et al. used the mouse model with inefficient Dicer1 allele mutation to study and found that the Dicer1 defect impaired the growth of new capillaries in the ovaries of female mice, resulting in luteal insufficiency and infertility in female mice (Otsuka et al., 2008). Further studies showed that impaired luteal angiogenesis in Dicer1-deficient female mice was partly caused by the deletion of miR-17-5p and let-7b. miR-17-5p and let-7b can participate in angiogenesis through targeted regulation of Tissue inhibitor of metalloproteinase 1 (Timp1) expression. In addition, intra ovarian injection of miR-17-5p and let-7b in Dicer1-deficient female mice could partially normalize Timp1 expression and luteal vascular growth, but could not sustain pregnancy. This may be due to the fact that many other miRNAs are required for the maintenance of pregnancy, or that the amount of miR-17-5p and let-7b injected into the ovary is insufficient or cannot exist for a long time (Otsuka et al., 2008). MiRNAs also played an important role in ovulation in mice. Oocyte specific removal of Dicer results in spindle disorder and chromosome aggregation, leading to stagnation in the first meiosis stage (Murchison et al., 2007; Tang et al., 2007). Degradation of many maternal transcripts is critical to the completion of meiosis in oocytes, and miRNAs may be involved in this degradation process (Murchison et al., 2007).

miRNAs also play an important role in follicular granulosa cells. Specific removal of Dicer1 in mouse follicular granulosa cells leads to increased consumption of primitive follicle banks, accelerated recruitment of early follicles and increased number of degenerating follicles in ovary, and changes in many follicle-development-related genes, Cyp17a1, Cyp19a1, ZPS (Zona Pellucida Glycoproteins), GDF9 and BMP15 (Lei et al., 2010). In addition, FSH was found to regulate the expression of miR-29a and miR-30d in cultured granulosa cells in a time-specific manner (Yao et al., 2010b). The appearance of LH peak can promote the expression of miR-21, miR-132 and miR-212 in granulosa cells (Fiedler et al., 2008). TGF-β1 induced the expression of miR-224 in cultured mouse granulosa cells (Yao et al., 2010a). After 12 h treatment with FSH, progesterone expression was promoted, and 17 up-regulated miRNAs and 14 down-regulated miRNAs (including miR-29a and miR-30d) were found, suggesting that miRNAs may be involved in mediating FSH regulation of gene expression and hormone production in granulosa cells (Yao et al., 2010b). Yao et al. treated primary cultured mouse granulosa cells with TGFβ1, and screened 13 down-regulated miRNAs and three up-regulated miRNAs. Up-regulated miR-224 participated in the regulation of TGFβ1 in granulosa cell proliferation and estrogen secretion by acting on Smad4, while regulating the expansion of the cumulus oophorus by acting on Ptx3 (Yao et al., 2010a; Yao et al., 2014). Further research shows that p53 and p65 may affect female reproduction by regulating the miR-224-smad4 pathway alone or synergistically (Liang et al., 2013).

## LncRNAs

### LncRNA Functions

**FIGURE 2:**
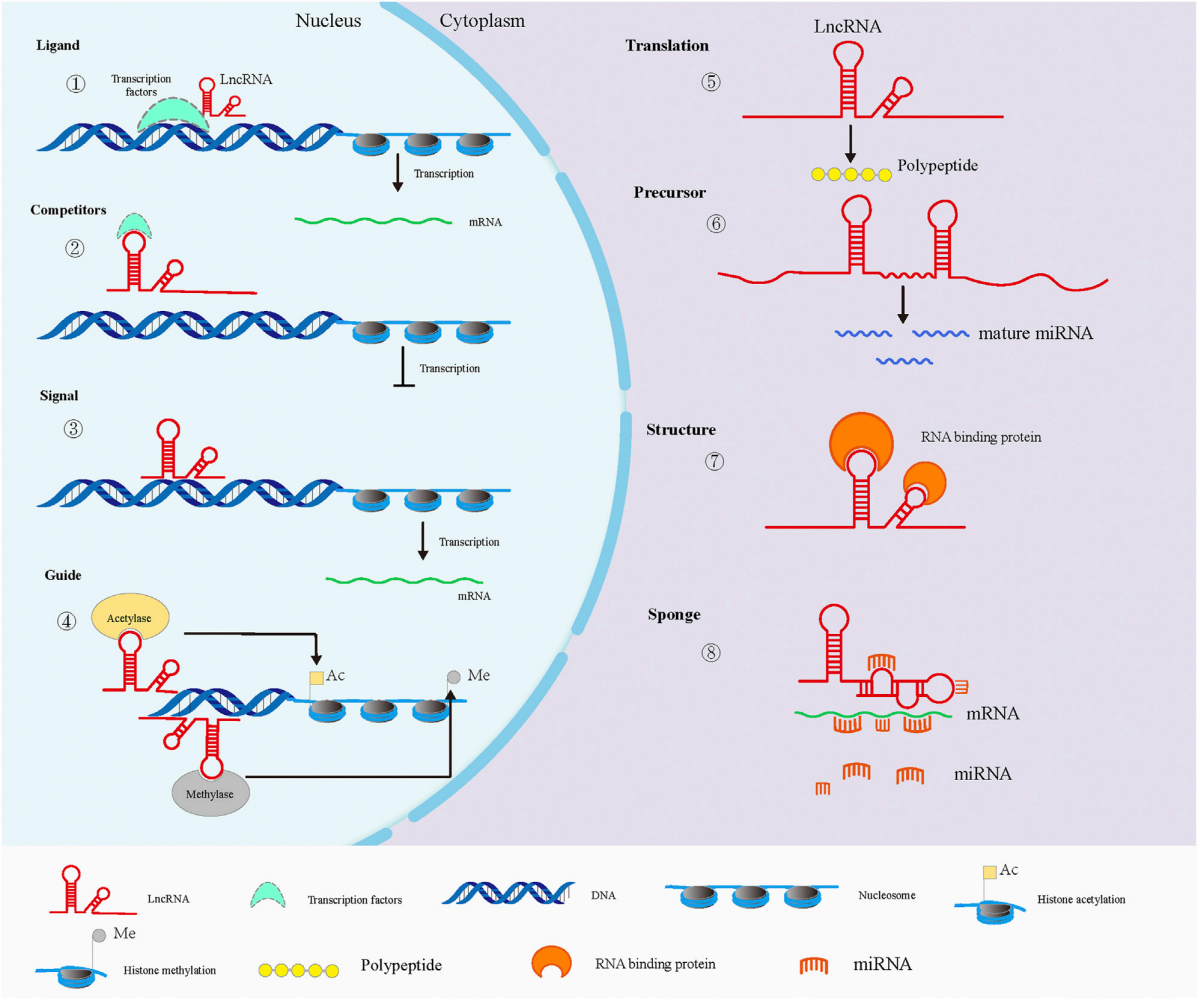
Main biological functions of lncRNAs. In the nucleus (left), lncRNAs can interact with transcription factors to promote (1) or inhibit (2) gene transcription. In addition, lncRNA can directly regulate gene transcription as a signaling molecule(3). LncRNAs can also recruit histone modifiation-related enzymes to participate in epigenetic regulation, including histone acetylation and methylation (4). In the cytoplasm (right), minority LncRNAs have some coding ability to translate peptides (5). LncRNAs are the source of some sncRNAs in the cytoplasm (6). Some RBPs can interact with lncRNAs via specific motifs (7). Besides, lncRNAs can alleviate the inhibition of miRNA on mRNA translation via sponging miRNAs (8).

Mammalian transcriptome analysis revealed that most lncRNAs are transcribed by RNA polymerase II, which is similar to protein-coding mRNA, with a 5 ′methylation cap and a polyadenylate tail but without a distinct open reading frame (Wilusz et al., 2009; Kopp and Mendell, 2018). Although lncRNA cannot encode protein, it can play a role in a variety of diseases through its own structural characteristics or as a signal molecule, such as neurological diseases, cardiovascular diseases, autoimmune diseases and tumors (Wapinski and Chang, 2011; Kwok and Tay, 2017; Distefano, 2018). The main biological functions of lncRNAs are shown in Figure 2. In the nucleus, some lncRNAs can be used as ligands of transcription factors to promote gene transcription, and can also negatively regulate the transcription process by competing transcription factors (Chen, 2016), and some lncRNAs can be used as signal molecules to regulate the transcription process (Guo et al., 2019). In addition, some specific lncRNAs can regulate gene expression by recruiting histone modification enzymes to specific sites and changing chromatin activity (Sun et al., 2016; Liu et al., 2017b). In the cytoplasm, a few lncRNAs with certain coding ability exert their functions by translating short peptides (Ruiz-Orera et al., 2014). Certain lncRNAs are precursors of miRNAs, and mature miRNAs can be generated after cleavage. Studies have also reported that lncRNAs can sponge miRNAs through the principle of base complementary pairing to inhibit the function of miRNAs. In addition, lncRNA can also combine with RBP to regulate the splicing, stability and initiation of translation of target mRNA, thus regulating gene expression in the translation stage (Yoon et al., 2014; Dykes and Emanueli, 2017).

### The Role of lncRNAs in Spermatogenesis

A large number of lncRNAs have been detected in the testes of humans, rats and mice during specific developmental stages; some of the functions of lncRNAs have been characterized and annotated, for instance, Mrhl (meiotic recombination hot spot locus) (Arun et al., 2012), Tsx (testis-specific X-linked gene) (Anguera et al., 2011), Dmr (Dmrt1-related gene) (Zhang et al., 2010) and HongrES2 (Ni et al., 2011) play an important role in testicular development and spermatogenesis. For instance, Testis-specific X-linked (Tsx), as a LncRNA, was specifically expressed in pachytene spermatocytes, testicular weight decreased and spermatocyte apoptosis was observed in the Tsx knockout mice.

SSCs are a uniquely male germ line stem cell, which can support spermatogenesis and maintain male fertility. Liang et al. found that 241 specific lncRNAs can play an essential role in maintaining SSC survival and self-renewal through protein coding genes and miRNA (Liang et al., 2014). Weng et al. used RNA sequencing and found that 564 lncRNAs were specifically identified from 60-day-old porcine testes vs 90-day-old, it was suggested that these lncRNAs might play a potential role in porcine SSC self-renewal (Weng et al., 2017). Many studies have confirmed that lncRNAs are the key regulator of SSCs, but their role in SSC-related physiological processes has only been partially studied. Ddx5/p68 has been found to be an interaction protein of lncRNA Mrhl in spermatogonial cells, and Mrhl is involved in nuclear reservation of p68. When Mrhl or p68 is knocked down, the nuclear localization of β-catenin, a key protein in the Wnt pathway, is severely affected. Further experiments verified that Mrhl expression was down-regulated when Wnt pathway was activated, indicating that Mrhl was a negative regulator of the Wnt pathway (Arun et al., 2012). Other research has also been reported that Mrhl can directly recruit Sin3a and HDAC1 at the Sox8 promoter site to form a coinhibitory complex, thus reducing the expression level of Sox8. During spermatogonial differentiation, the Wnt pathway is activated and this inhibition disappears. These results suggest that Mrhl also plays an important role in meiosis entry and spermatogonial differentiation (Kataruka et al., 2017). Hu et al. found that the lncRNA Gm2044 could inhibit the proliferation of GC-1 cells (mouse spermatogonia cell line) by binding to UTF-1 (Undifferentiated embryonic cell transcription factor 1). Higher Gm2044 expression levels in GC-1 cells resulted in increased cell viability and UTF1 protein inhibition. The lncRNA Gm2044 is also highly expressed in pachytene spermatocytes and inhibits Utf1 mRNA expression during spermatogenesis (Hu et al., 2018).

In view of the fact that a large number of lncRNAs have been found to be expressed in testicular development and male germ cells, but only a few have studied their functions during the development of male germ cells, the regulatory mechanism of lncRNA in spermatogenesis is worth further in-depth investigation.

### The Role of lncRNAs in Follicular Development

Cumulus cells (CCs) are closer to oocytes than parietal granule cells, participate in the coordination of late follicular development and oocyte maturation, promote amino acid transport, sterol biosynthesis and glycolysis, and provide energy substrates for oocyte meiosis recovery (Hillensjo et al., 1982). Xu et al. retrospectively analyzed and compared the CC gene expression profiles of the high-quality embryo group and poor-quality embryo group according to clinical embryo development. The results showed that lncRNA Y00062, which was significantly down-regulated in CCs of the poor-quality embryo group, may be involved in regulation of the NEK7 gene (Xu et al., 2014). NEK7 is a gene that is essential for spindle assembly and mitosis (Gupta et al., 2017), indicating that the lncRNA Y00062 can play an important role in regulating the meiosis of oocytes. The lncRNA ENST00000502390 is highly expressed in CCs derived from poor-quality embryos and is related to ELOVL5 (elongation of very-long-chain fatty acids protein 5). ELOVL5 is associated with the biosynthesis of highly unsaturated fatty acids and regulates the maturation and ovulation of oocytes. That is, the lncRNA ENST00000502390 may regulate the synthesis of highly unsaturated fatty acids, and participate in oocyte maturation and ovulation (Warzych et al., 2017).

In ovarian granule cells of patients with PCOS, the lncRNA HCG26 (HLA complex group 26) is highly expressed and its expression level is linked to the number of ovarian antral follicles. The down-regulation of HCG26 expression in the human ovarian granule cell line KGN can inhibit cell proliferation and cell cycle progression, and also affect the expression of the aromatase gene and the production of 17β-estradiol (Liu et al., 2017a). It is suggested that HCG26 may be an essential regulatory gene affecting granule cell proliferation and follicular growth in patients with PCOS. It has been reported that the lncRNA Gm2044 acts as a competing endogenous RNA for miR-138-5p to rescue the inhibitory effects of miR-138-5p on Nr5a1, and then regulate 17β-estradiol synthesis (Hu et al., 2019). This serves as a new focal point for the treatment of PCOS. LncRNA Amhr2 was transcribed upstream from Amhr2 gene which regulated of anti-Mullerian hormone specific effects in ovarian granulosa cells. It was proved that LncRNA Amhr2 enhanced the promoter activity of Amhr2 and significantly inhibited the expression of Amhr2 after knocking down lncRNA Amhr2 in primary granulosa cells (Kimura et al., 2017).

## PIWI-Interacting RNA

### PIWI-Interacting RNA Functions

In 2006, four separate teams identified a new class of small RNA molecules, called PIWI-interacting RNAs, that specifically interact with PIWI proteins in the germline cells of fruit flies, mice, rats and humans. hereinafter referred to as piRNA (Aravin et al., 2006; Girard et al., 2006; Grivna et al., 2006; Lau et al., 2006). piRNAs are an animal-specific kind of small silencing RNAs which characteristic expression in pachytene spermatocytes and sperm cells during spermatogenesis (Gou et al., 2015). piRNAs interact with the PIWI protein to play a role in spermatogenesis. An increasing number of studies have shown that subfamily PIWI proteins, including MIWI, MIWI2 and MILI*,* are essential factors for stem cell regeneration and male germ cell development in vertebrates (Reddien et al., 2005; Klattenhoff and Theurkauf, 2008).

The expression of three PIWI proteins in mice showed a strict temporal sequence during spermatogenesis: MILI was the first to be expressed at 12.5 d of embryo stage and continued to the spherical sperm cell stage after the completion of meiosis; The expression of MIWI2 began at 15.5 days of embryo stage and ended at 3 days after birth, which was the shortest expression time of Piwi protein. The last is MIWI, whose expression begins in the fine line phase of meiosis and ends in late sperm cells (Siomi et al., 2011). Correspondingly, piRNA expression also appeared two peaks before and after birth and at meiosis stage, piRNA of prepachytene and piRNA of pachytene, respectively (Aravin et al., 2008). Prepachytene piRNA was mainly derived from transposable element sequence and combined with MILI and MIWI2. Pachytene piRNA comes from intergene, intron and exon regions and mainly binds to MILI and MIWI. Different Piwi proteins in mice bound piRNA of different lengths: MILI bound piRNA of (∼26 nt) was the shortest. MIWI2 combined with (∼28 nt) piRNA; The piRNA combined with MIWI was the longest (∼30 nt) (Siomi et al., 2011). On the one hand, the expression and function of Piwi/piRNA are necessary for sperm cell development and differentiation. On the other hand, the composition of Piwi/piRNA functional complex also showed space-time specific dynamic changes during spermatogenesis.

### The Role of PIWI-Interacting RNA in Spermatogenesis

Previous studies have shown that Piwi/piRNA mainly silences transposition elements and regulates the expression of protein-coding genes at the epigenetic and post-transcriptional levels, thus playing an important role in the development and differentiation of animal germ cells and gametogenesis (Ozata et al., 2019). Transposon can be inserted into other sites in the genome by transcription or reverse transcription, and under the action of endonuclease, thus affecting the stability and integrity of the genome (Onishi et al., 2021). Therefore, the activity of transposable elements needs to be strictly controlled.

Before and after mouse birth, MILI protein can bind piRNA derived from transposon and then cut transposable element RNA which is homologous and complementary to piRNA to generate secondary piRNA. The newly generated secondary piRNA can also bind to MILI and also cut the same homologous and complementary transposable element RNA with secondary piRNA to generate a new primary piRNA. If the reciprocating cycle, namely ping-pong cycle, can continuously cut the RNA transcribed from transposable element, thus effectively silencing transposable element activity (Onishi et al., 2021). Defects in cleavage activity of MIWI and MILI proteins lead to activation of transposable elements such as LINE-1, It is suggested that MIWI and MILI proteins may directly clew transposable element RNA in male germ cells of mice (De Fazio et al., 2011; Reuter et al., 2011).

More and more experimental evidence indicates that in addition to transposon silencing, piRNA can also play a biological role by regulating the expression of protein-coding genes. In mouse elongate sperm cells, pachytene piRNA, its binding protein MIWI and deadenylase CAF1 form a Pi-RISC complex, which recognizes the sequence elements of the 3 ′ untranslated region (3′ UTR) of target mRNA by a base incomplete pairing approach similar to miRNA. Inducing deadenylation and degradation of target mRNA, and depending on the different sequences of piRNA in millions, pi-RISC mediates degradation of thousands of different mRNA during late sperm cell development (Gou et al., 2015). In addition, pachytene piRNA can also guide MIWI protein to cleavage and degrade target mRNA in testicular tissue through a siRNA-like mechanism when it is fully or nearly fully paired with target mRNA (Zhang et al., 2015). Besides, the proper removal of MIWI/piRNA was crucial for sperm maturation in late spermatogenesis. piRNAs regulated the clearance of MIWI/piRNA machine through ubiquitin-proteasome signaling pathway, which further confirms that piRNA plays a very considerable role in the development of male germ cells (Zhao et al., 2013). Interestingly, piRNA not only targets mRNA degradation, but also promotes mRNA translation. It has been reported that MIWI/piRNA, in conjunction with eIF3f and HuR, can regulate the translation of a large number of mRNA in sperm cells, and this mechanism has been proved to be necessary for the successful acrosome assembly in mouse spermatogenesis (Dai et al., 2019). In this study, MIWI/piRNA mediated translation activation in sperm cells, providing a new clue to the important biological problem of transcription-translation uncoupling during sperm formation.

### The Role of PIWI-Interacting RNA in Follicular Development

In recent years, researchers have conducted in-depth studies and found that piRNA is not only specifically expressed in testis, but also abundant piRNAs are detected in ovary (Williams et al., 2015). It has been reported that PIWIL2 was expressed in human fetal oocytes and PIWIL1 and PIWIL2 in adult ovaries. Bovine ovaries expressed PIWIL1 and high expression PIWIL3 was isolated from bovine oocytes. PiRNA is also abundant in human, bovine and rhesus monkey oocytes, but their function in oocytes has not been clearly studied (Roovers et al., 2015; Williams et al., 2015). It was found that PIWI protein was also expressed in mouse ovary oocytes, but the loss of PIWI protein did not affect female reproduction. Recent studies have reported that endo-siRNA similar to siRNA is highly expressed in mouse oocytes, while most mammalian oocytes, including human and monkey oocytes, do not express endo-siRNA (Zhang et al., 2021). Because the types and expression characteristics of small RNA in golden hamster oocytes are similar to those of most other mammals, including humans and cynomolgus monkeys, golden hamster models of PIWIL1 deletion mutants were established. Interestingly, PIWIL1-deficient females had normal ovaries and were able to produce mature oocytes, but the fertilized eggs produced by mating with wild-type males did not develop properly and remained stuck in the two-cell stage. It was found that the deletion of PIWIL1 gene resulted in abnormal accumulation of transposons in oocytes, decreased degradation of maternal mRNA and failure of zygotic gene activation during early embryonic development (Hasuwa et al., 2021; Loubalova et al., 2021). These results suggest that piRNA pathway may also play an important role in human and other mammalian female reproduction, providing an important theoretical basis and animal model for the diagnosis and treatment of piRNA pathway abnormalities and female reproductive diseases.

## Non-Coding RNA in Reproductive Diseases

### The Role of Non-Coding RNA in Male Reproductive Diseases

Currently, infertile couples account for about 15% of married couples worldwide, and male infertility accounts for 40–50% (Dada et al., 2003). The etiology of infertility is complex, and the detection methods and related research are still relatively sparse. Research into male infertility from the molecular perspective has become important, which opens up new fields for analysis of the etiology of infertility (Robles et al., 2019).

A study using miRNA chip technology to screen the semen of infertile and healthy men found that there were 52 differentially expressed miRNAs, of which 21 were highly expressed miRNAs and 31 showed low expression in infertile patients (Liu et al., 2012). Using the same technique in normal testicular tissue and non-obstructive azoospermia patients, it was found that the expression of 173 miRNAs was altered in infertile patients, with 19 miRNAs showing up-regulated expression and 154 miRNAs being down-regulated (Zhuang et al., 2015). It was also shown that mir-34c and mir-275 were significantly and highly expressed in the testicles of NOA patients, while studies found that the highly expressed miR-34c could initiate germ cell apoptosis by targeting the amp-dependent periodic transcription factor ATF1 (Liang et al., 2012). Studies have shown that miRNA-122-5p expression is up-regulated in the testicular tissue of patients with obstructive azoospermia (OA) compared with those with non-obstructive azoospermia (NOA). miRNA-122-5p enhanced SSC cell activity by competitively binding with the lncRNA CASC7 and targeting CBL mRNA (Zhou et al., 2020). Due to the significantly differential expression of miRNA in NOA patients and its important biological functions, miRNAs can be used as a potential molecular marker for the diagnosis and typing of male fertility, and can also provide a new solution for the clinical treatment of male infertility according to the function of miRNAs in spermatogenesis.

At the gene level, lncRNAs are not separated from DNA methylation. However, this model is commonly seen in the combination of lncRNA and methylated transferase (DNMT1, 3, etc.), and the localization of this enzyme to the promoter (CpG island) and methylation of the gene, thus inhibiting gene transcription (Somasundaram et al., 2018; Kang et al., 2019). Peng et al., analyzed DNA methylation levels in sterile and normal male sperm and found that abnormal methylation of imprinting genes H19 and SNRPN was related to abnormal sperm parameters and male infertility (Peng et al., 2018). Additional studies have found that DNA methylation mediates male reproductive organ development, spermatogenesis and male sexual behavior by affecting gene expression, indicating that lncRNAs are closely related to male infertility (Cisneros, 2004).

### The Role of Non-Coding RNAs in Female Reproductive Diseases

PCOS (Polycystic ovary syndrome) is the principal element of female infertility, and is often characterized by hyperandrogenemia, oligomenorrhea and polycystic ovary syndrome, affecting 6–10% of women worldwide (Bozdag et al., 2016). In addition, about 90% of an ovulatory infertility is caused by PCOS (Misso et al., 2012). The etiology and pathogenesis of PCOS remain unclear, there is no effective cure, clinical treatment is mainly symptomatic, and long-term health management is needed. In recent years, many studies have found that non-coding RNAs play a significant role in PCOS. However, the research into the role of lncRNAs in the pathogenesis of PCOS is sparse. This paper mainly reviews the research advances of lncRNAs in PCOS.

With the development of gene sequencing technology, countless researchers have carried out the study of lncRNA sequencing in PCOS. The researchers completed microarrays or deep sequencing of different ovarian cells from PCOS patients or a rat model (Huang et al., 2016; Liu et al., 2017a; Fu et al., 2018a). These results describe hundreds of thousands of differentially expressed lncRNAs. TERRA is a lncRNA composed of different numbers of UUAGGG repetitive sequences, (Azzalin et al., 2007). TERRA is associated with the regulation of telomere length, telomerase activity and heterochromatin (Wang et al., 2015). Some studies have suggested that abnormalities of telomeres may be involved in the pathogenesis of PCOS (Wei et al., 2017). Wang et al. performed a prospective control study and found that the relative telomere length of PCOS patients was higher than that of the control group, but the expression level of TERRA was lower than that of the control group. In the PCOS group, the expression level of TERRA was negatively correlated with the concentration of testosterone, while the telomere length of leukocytes was positively correlated with the concentration of testosterone (Wang et al., 2017a), suggesting that TERRA and testosterone regulate the telomere length of leukocytes, resulting in PCOS.

In addition, many lncRNAs are involved in the occurrence and development of PCOS, lnc-MAP3K13-7:1 was highly expressed and accompanied by DNA hypommethylation in granulosa cells of PCOS patients. The study demonstrated that Lnc-MAP3K13-7:1 bound DNMT1 as a recruitment agent and mediated its ubiquitination degradation. The CDKN1A promoter was demethylated and its expression level increased, finally, GCs proliferation was inhibited (Geng et al., 2021). CTBP1-AS is a lncRNA associated with the androgen receptor signaling pathway. Liu et al. found that after adjusting for age and body mass index (BMI), the expression of CTBP1-AS in peripheral blood leukocytes of women with PCOS was higher than that of women in the control group, and showed that individuals with a high expression of CTBP1-AS had an increased risk of PCOS. After adjusting for age, BMI and IR to evaluate the steady-state model, the expression level of CTBP1-AS was positively correlated with the concentration of serum total testosterone (Liu et al., 2015).

Research into the role of lncRNAs in PCOS has just begun. Although the sequencing results show that there are numerous differentially expressed lncRNAs, research into these differentially expressed lncRNAs is not prolific enough, and only a few studies have discussed the function and mechanism of lncRNAs. Current studies mainly focus on the expression and mechanism of lncRNAs in peripheral blood leukocytes, cumulus cells, granulosa cells and follicular fluid. According to the current research, lncRNAs may participate in the production of steroids, enhance the activity of steroid receptors, obesity and IR and other metabolic processes, affect the development, proliferation and apoptosis of ovarian cells, and then lead to the development of PCOS (Chen et al., 2019b; Mai et al., 2019). The study of lncRNAs in PCOS has a broad prospect, and more extensive and in-depth studies are needed to explore the mechanism of action of lncRNAs in PCOS and the relationship between lncRNAs and miRNAs, in order to help us to better understand the pathogenesis of PCOS; this is of great significance for the clinical diagnosis and treatment of this syndrome.

In recent years, more and more studies have investigated miRNA expression in women with PCOS (Santamaria and Taylor, 2014; Butler et al., 2020; Gebremedhn et al., 2021). It has been reported that there are a variety of miRNAs in human follicular fluid, including miR-483-5p, miR-132, miR-320, miR-520c-3p, miR-24 and miR-222, which are related to estrogen secretion. miR-24, miR-193b and miR-483-5p regulate progesterone secretion, and the expression of miR-132 and miR-320 in follicular fluid of polycystic follicles is significantly lower than that in normal follicular fluid (Sang et al., 2013; Sorensen et al., 2016). These results suggest that miRNA in follicular fluid plays an important role in regulating steroid hormone synthesis. Changes in their expression are associated with polycystic ovaries. Roth et al. compared miRNAs in follicular fluid of patients with normal follicles and polycystic ovary, and identified 5 miRNAs that were up-regulated in follicular fluid of polycystic ovary. They are miR-9, miR-18b, miR-32, miR-34c and miR-135a, respectively, and their potential target genes interleukin-8 (IL-8), Synaptogamin1 and insulin receptor substrate2 (IRS2) are down-regulated in polycystic ovaries (Roth et al., 2014). The abnormal expression of these genes is closely associated with abnormal follicular development and polycystic ovaries. In addition, up-regulated miR-27a-3p inhibits granulosa cell proliferation by targeting Smad5 and Creb1 genes, promotes apoptosis of ovarian granulosa cells and transformation of androgen and estrogen, thus participating in the occurrence of abnormal PCOS follicles (Wang et al., 2017b; Wang et al., 2018a). Besides, some miRNAs that function in the reproductive system are summarized in Table 1.

**TABLE 1:** Function of lncRNAs in reproductive system.

Gene name	Species and samples	Biological functions	Mechanism	Reference
LncRNA HOTTIP	Mouse spermatogonial cell line GC-1 cells	Promote cells G2/M phase arrest and early apoptosis	The expression of γ -H2Ax and p53 was up-regulated by adjacent gene Hoxa13	Liang and Hu (2019)
NONMMUT074098.2	Mouse spermatogonial cell line GC-1 cells	Apoptosis	Knocking down NONMMUT074098.2 activated the p38 MAPK pathway	Li et al. (2019b)
LncRNA AK015322	Mouse SSC line C18-4 cells	Promote cells proliferation	LncRNA AK015322 sponged miR-19b-3p and alleviated the inhibition of ETV5	Hu et al. (2017)
LncRNA ZFAS1	Human ovarian granulosa cells(PCOS patients)	Endocrine, proliferation and apoptosis	LncRNA ZFAS1 bound miR-129 to promote the expression level of HMGB1	Zhu et al. (2020)
LncRNA NEAT1	Ovarian granulosa cells (PCOS rats)	Proliferation and apoptosis	Knockdown LncRNA NEAT1 promoted IGF1 expression via miR-381	Zhen et al. (2021)
LncRNA ROR	Human epithelial ovarian carcinoma cell lines SKOV3 and 3AO cells	Epithelial-mesenchymal transition (EMT)	LncRNA ROR promoted EMT via mir-145/FLNB axis	Li et al. (2019a)
LINC00339	Human epithelial ovarian carcinoma cell lines SKOV3 and HO-8910 cells	Proliferation, migration, and invasion	LINC00339 bound miR-148a-3p to promote the expression level of ROCK1	Pan et al. (2019)
LncRNA SLC7A11-AS1	Human testicular embryonic carcinoma cells line NCCIT cells	Increased ROS levels	The expression of SLC7A11 was negatively regulated and the oxidative stress level of cells was increased	Sanei-Ataabadi et al. (2020)
miR-18b	Human follicular fluid with PCOS	Promoted progesterone release, inhibited testosterone and estradiol release	miR-18b targeted the mRNAs of IL8, SYT1 and IRS2	Roth et al. (2014)
miR-224	Mouse granulosa cells	Induced GCs proliferation and increased estrogen release	miR-224-mediated target gene Smad4 pathway	Yao et al. (2010a)
miR-16	Human ovarian granulosa cells with PCOS	Proliferation cycle and apoptosis	miR-16 promoted the proliferation and inhibited apoptosis of ovarian GCs by directly targeting PDCD4	Fu et al. (2018b)
miR-141, miR-429 and miR-7-1-3p	Human semen with non-obstructive azoospermia patients	Potential markers of NOA	miR-141 reduced the expression levels of Cbl and Tgfβ2, and miR-7-1-3p down-regulated Rb1 and Pik3r3.	Wu et al. (2013)

Ovarian cancer constitutes one of the most common cancers of the female reproductive system. However, the molecular mechanism of ovarian cancer remained unclear, so it is necessary to further study the biological behavior of ovarian cancer at present. NcRNAs provided a suitable cut-off point in the diagnosis and treatment of ovarian cancer. Previous studies have revealed that microRNA-224 inhibits the expression of cyclin A by targeting KLLN, thereby promoting the proliferation of epithelial ovarian cancer cells (Hu and Liang, 2017). In addition, we identified some lncRNAs that regulate reproductive system functions, which are detailed in Table 1.

## Outlook

Non-coding RNAs are important regulatory molecules in the human body and are essential for the development of a pathophysiological state. The formation of gametes is extremely composite, and spermatogenesis can be simply summarized as the proliferation and meiosis of primitive germ cells, with the formation of mature sperm occurring due to the deformation of sperm cells. The development of follicles requires the passage of primordial follicles, primary follicles, secondary follicles, and mature follicles, and the eventual discharge of the cumulus oocyte complex. These processes are strictly and precisely regulated, and the failure of any one of them can intervene in the continuation of life. Non-coding RNAs are an important element in these processes.

Despite the fact that current high-throughput experimental techniques and open ncRNA database resources have given rise to the discovery of many ncRNAs, the function of these ncRNAs in gametogenesis and reproductive disease remains unclear. Therefore, it can be inferred that there are still innumerable reproductive ncRNAs with important functions to be studied. This is also suggested that we have great potential to study the regulatory mechanism of ncRNAs in gametogenesis and reproductive disease. MiRNA binding to mRNAs is reversible, enabling the regulation of gene expression. Competitive endogenous RNA (ceRNA) competitively binds miRNA to regulate gene expression. LncRNAs can act as a sponge of miRNAs, adsorbing miRNAs and interacting with each other to constitute a huge and complex ceRNA network which is able to regulate the function of target genes.

Research into the role and mechanism of ncRNAs in the generative process is a difficult and persistent task. Understanding the role of post-transcriptional gene regulation in reproduction will help to clarify the causes of reproductive failure and hopefully provide methods and targets for the treatment of infertility, as well as additional contraceptive methods. Scientists need to continue to clarify the functions of lncRNA in spermatogenesis and oogenesis, and strive for the diagnosis and treatment of infertility.
